# Development of novel machine learning model for right ventricular quantification on echocardiography—A multimodality validation study

**DOI:** 10.1111/echo.14674

**Published:** 2020-05-12

**Authors:** Ashley N. Beecy, Alex Bratt, Brian Yum, Razia Sultana, Mukund Das, Ines Sherifi, Richard B. Devereux, Jonathan W. Weinsaft, Jiwon Kim

**Affiliations:** ^1^ Greenberg Cardiology Division Department of Medicine Weill Cornell Medicine New York NY USA; ^2^ Department of Radiology Mayo Clinic (Minnesota) Rochester MN USA

**Keywords:** echocardiography, right ventricle, right ventricular function

## Abstract

**Purpose:**

Echocardiography (echo) is widely used for right ventricular (RV) assessment. Current techniques for RV evaluation require additional imaging and manual analysis; machine learning (ML) approaches have the potential to provide efficient, fully automated quantification of RV function.

**Methods:**

An automated ML model was developed to track the tricuspid annulus on echo using a convolutional neural network approach. The model was trained using 7791 image frames, and automated linear and circumferential indices quantifying annular displacement were generated. Automated indices were compared to an independent reference of cardiac magnetic resonance (CMR) defined RV dysfunction (RVEF < 50%).

**Results:**

A total of 101 patients prospectively underwent echo and CMR: Fully automated annular tracking was uniformly successful; analyses entailed minimal processing time (<1 second for all) and no user editing. Findings demonstrate all automated annular shortening indices to be lower among patients with CMR‐quantified RV dysfunction (all *P* < .001). Magnitude of ML annular displacement decreased stepwise in relation to population‐based tertiles of TAPSE, with similar results when ML analyses were localized to the septal or lateral annulus (all *P* ≤ .001). Automated segmentation techniques provided good diagnostic performance (AUC 0.69–0.73) in relation to CMR reference and compared to conventional RV indices (TAPSE and *S*′) with high negative predictive value (NPV 84%–87% vs 83%–88%). Reproducibility was higher for ML algorithm as compared to manual segmentation with zero inter‐ and intra‐observer variability and ICC 1.0 (manual ICC: 0.87–0.91).

**Conclusions:**

This study provides an initial validation of a deep learning system for RV assessment using automated tracking of the tricuspid annulus.

## 1 INTRODUCTION

Right ventricular (RV) dysfunction is a well‐established prognostic marker for a wide range of conditions including pulmonary hypertension, cardiomyopathy, and congenital heart disease.^1^, ^2^, ^3^ Echo is the most widely used screening tool to assess the RV with methodologies studied in relation to reference standards and for prediction of cardiovascular outcomes.^4^, ^5^ However, challenges for RV functional assessment by echo are well documented primarily due to complex RV geometry.^6^, ^7^ Moreover, current 2D methodologies for RV assessment require additional M‐mode and tissue Doppler velocity image acquisition and analysis for which accuracy is dependent on an on‐axis cursor placement in the direction of tricuspid annular displacement.

Machine learning (ML)–based methodologies have the potential to provide fully automated image analysis. Conventional neural networks–based segmentation techniques have been applied to echo, though with focus primarily on left ventricular chamber size and systolic function quantification.^8^ While a recent study examined a ML approach for three‐dimensional echocardiography (3DE) assessment of RV volume and EF,^9^ limited clinical availability of 3DE is a known barrier for widespread utilization. Fully automated ML approaches have yet to be applied for RV assessment on standard 2D echo and have the potential to improve efficiency and accuracy without need for additional M‐mode, tissue velocity, or 3D image acquisition.

This study examined RV functional assessment using a novel ML‐derived fully automated approach for RV quantification on routine 2D echo. The primary study aim was to determine the feasibility and reproducibility of automated ML algorithm for cardiac magnetic resonance (CMR) quantified RV dysfunction among a prospectively enrolled cohort of patients undergoing echo and CMR.

## 2 METHODS

### 2.1 Study population

The study population comprised prospectively enrolled patients with known or suspected coronary artery disease (CAD) between September 2015 and December 2018 in a multimodality imaging protocol focused on cardiac chamber remodeling. Patients underwent echo and CMR within a narrow interval (99% the same day). Patients with contraindications to contrast‐enhanced CMR (eg, GFR < 30 mL/min/1.73 m^2^, ferromagnetic implants) were excluded. In all patients, comprehensive demographic data were collected using standardized questionnaires, including cardiac risk factors and medications. This study was conducted with approval of the Weill Cornell Medical College Institutional Review Board, which was in compliance with the Declaration of Helsinki; written informed consent was obtained at time of enrollment.

### 2.2 Imaging protocol

Echo and CMR were performed using a standardized image acquisition protocol:

#### 2.2.1 CMR

CMR was performed using 3.0 Tesla scanners (General Electric). Cardiac chamber volumes were assessed via cine‐CMR (steady‐state free precession), which included long‐axis^2^, ^3^, ^4^ as well as contiguous short‐axis slices acquired from the tricuspid valve annulus through the RV apex that were quantified at end‐diastole and end‐systole for calculation of RV ejection fraction (RVEF). CMR RV_DYS_ was defined as RVEF < 50%.

#### 2.2.2 Echocardiography

Transthoracic echoes were acquired using commercial equipment (Philips iE33). Echoes were interpreted by experienced investigators within a high‐volume laboratory for which expertise and reproducibility for quantitative RV indices have been previously reported.^5^ RV function was quantified using tricuspid annular plane systolic excursion (TAPSE) and RV systolic excursion velocity (*S*′). Measurements were acquired in accordance with American Society of Echocardiography (ASE) guidelines; established cutoffs (TAPSE < 1.6 cm, *S*′ < 9.5 cm/s) were used to define RV_DYS_ by each parameter.^10^ Echo analyses were performed blinded to CMR results.

### 2.3 Image processing

Manual segmentation maps were created by annotating the free‐wall (lateral) and septal tricuspid annulus in each echo frame (n = 7791 frames) within the echo examinations (n = 101) with a small circular segmentation map of uniform size. Segmentation entailed labeling pixels in the magnitude images using 3DSlicer, an open‐source medical image postprocessing application.^3^ Manual segmentation was performed by an experienced (level III trained) physician (JK). Inter‐reader reproducibility for manual segmentation was determined via analysis of a random subset of 22/101 (22%) of studies.

Images were resampled and (if necessary) zero‐padded to 256 × 256 pixels. Pixel intensity values were then rescaled to values between zero and one. Aggressive data augmentation was employed in the form of random zoom, rotation, crop (224 × 224), horizontal/vertical flip, and addition of Gaussian noise.

### 2.4 Model and training

**FIGURE 1:**
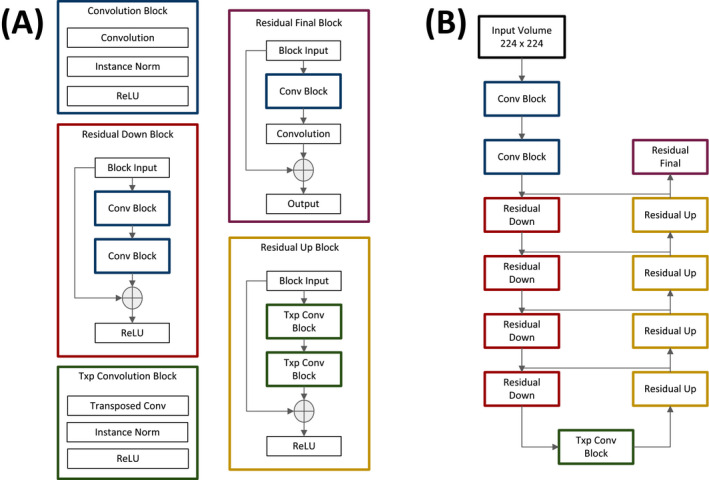
Network modules (A) and architecture schematic (B). Design of the network based on the U‐net architecture, which is widely used in medical segmentation tasks. Residual modules were employed to facilitate gradient flow during training and to prevent exploding/vanishing gradients. Horizontal lines between the contracting and expanding pathways of the network in (B) represent concatenation. Conv block = convolutional block; ReLU = rectified linear unit; Txp = transposed; Txp Conv Block = transposed convolutional block

The automated segmentation model was based on neural network architecture described by Han,^11^ a modified U‐net,^12^ for which excellent performance has been previously demonstrated in medical segmentation. The model makes use of residual modules,^13^ which improve gradient flow between adjacent layers and increase classification accuracy. A diagram of the model's architecture is shown in Figure 1.

The ML algorithm was initially trained and tested using sixfold cross‐validation. Cross‐validation is a procedure whereby data are randomly split into nonoverlapping subsets such that a model can be trained on all but one subset and tested on the remaining subset._ _In this case, a different model instance was trained and tested for each of the six holdout subsets and test metrics were averaged per‐case for the entire dataset. As is typical of cross‐validation, no model instance was tested on data from which it was trained. Cross‐validation was chosen in place of splitting into solitary train, validation, and test subsets because it better demonstrates the true performance of a model under a size‐constrained dataset. To minimize the risk of overfitting, neural network architecture, hyperparameters, cross‐validation groupings, and training protocols were not modified in any way after the model was exposed to the cross‐validation dataset. Modification of these parameters could result in improved measures of accuracy but this could be at the expense of generalizability.

A weighted softmax/cross‐entropy loss function was used for training as follows:

$$\text{Loss}\;\left(x,i\right)\;=\;-W\left[i\right]\;*\;\ln\;\left(e^{x[i]}/\Sigma jCe^{x[j]}\right)$$

where *x *is the output logit vector at a given pixel, *i *the true class label, *w *the vector of class weights, and *C* the number of classes. Weighting was employed to combat class imbalance given that the vast majority of pixels in each image were nonannular. A class weight of 0.2 was empirically assigned to the nonannular class and 0.8 to the annular class. RMSProp was used to apply incremental parameter updates.

The following automated and manual indices were derived using a segmentation map: (a) linear tricuspid annular displacement (LTAD), which corresponded to the maximum displacement between two positions of the tricuspid annulus, and (b) circumferential tricuspid annular displacement (CTAD), which represented the total bidirectional circumferential distance traversed by the annulus. LTAD and CTAD were calculated for both the lateral tricuspid annulus and the septal tricuspid annulus. All distances were calculated by considering the center of mass of all pixels containing an annular label as the position of the annulus. Annular motion was smoothed with a median filter.

The model was built in Python using the deep learning framework PyTorch. Training and testing were performed on a workstation with four CPU cores, 64 GB of system memory, and a graphics‐processing unit (GPU) with 11 GB of video memory (NVIDIA [Santa Clara] GTX 1080 Ti). Software code pertaining to both training and testing of the ML model can be found on line at:https://github.com/akbratt/RVTracker.

### 2.5 Model performance

The model was evaluated by comparing values of maximal displacement obtained from the automated segmentation compared to the manual segmentation. Measurements obtained from automated segmentation maps were compared to standard echo indices and RVEF on CMR, defined as the reference standard of RV functional assessment.

### 2.6 Statistical methods

Comparisons between groups were made using Student's *t* test (expressed as mean ± SD) for continuous variables. Inter‐ and intra‐observer agreement between methods was assessed using the method of Bland and Altman,^14^ which yielded mean difference as well as limits of agreement between methods (mean ± 1.96 SD). Bivariate correlation coefficients, intra‐class correlation coefficients, and linear regression equations were used to evaluate associations between variables. Statistical calculations were performed using SPSS 24.0 (Statistical Package for the Social Sciences, International Business Machines, Inc), SciPy,^15^ and Excel (Microsoft Inc). Two‐sided *P* < .05 was considered indicative of statistical significance.

## 3 RESULTS

### 3.1 Clinical application

**FIGURE 2:**
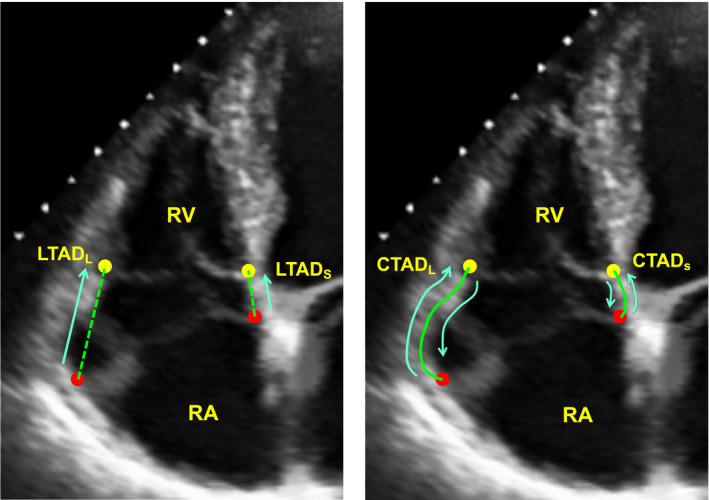
Annular displacement measurements. Representative lateral and septal LTAD (left) and lateral and septal bidirectional CTAD (right). Segmentation performed both manually and by machine learning algorithm. CTAD = circumferential tricuspid annular displacement; LTAD = linear tricuspid annular displacement; RA = right atrium; RV = right ventricle

Tricuspid annular shortening indices for RV functional assessment via manual and automated ML segmentation were tested in 101 patients equating to 7791 frames, among whom nearly one third (31%) had RV dysfunction (RVEF < 50%) as defined by CMR reference standard. Table 1 details clinical characteristics of the population, including comparisons between patients with and without CMR‐evidenced RV dysfunction. Segmentation via ML was successful in all cases, requiring minimal processing time (<1 second for all cases) and required no additional user editing. Figure 2demonstrates representative lateral and septal annular displacement performed both manually and by ML algorithm.

**TABLE 1:** Clinical characteristics

	Overall (n = 101)	RV dysfunction+ (n = 31)	RV dysfunction‐ (n = 70)	*P*
Clinical				
Age (y)	68 ± 10	69 ± 11	67 ± 10	.38
Male gender	81% (82)	87% (27)	79% (55)	.31
Body surface area	1.9 ± 0.2	1.9 ± 0.2	2.0 ± 0.3	.44
Coronary artery disease risk factors				
Hypertension	80% (81)	68% (21)	86% (60)	.04
Hypercholesterolemia	77% (78)	68% (21)	81% (57)	.13
Diabetes mellitus	51% (51)	58% (18)	47% (33)	.31
Tobacco use	62% (63)	68% (21)	60% (42)	.46
Family history	25% (25)	23% (7)	26% (18)	.74
Prior myocardial infarction	54% (54)	61% (19)	50% (35)	.29
Prior coronary revascularization	75% (76)	81% (25)	73% (51)	.40
Percutaneous intervention	55% (56)	61% (19)	53% (37)	.43
Coronary artery bypass	32% (32)	32% (10)	31% (22)	.93

Note

Data presented as mean ± SD (data in parentheses refer to range for each respective variable). RV dysfunction: RVEF < 50%.

Table 2 demonstrates three RV annular shortening quantification methodologies: manual segmentation, fully automated ML algorithm, and conventional RV indices. Findings demonstrate all annular shortening indices whether performed manually or by ML algorithm to be impaired among patients with RV dysfunction (*P* < .001 for all) with similar decrements regardless of quantification in the septal versus lateral tricuspid annulus. These results paralleled conventional annular displacement indices as evidenced by similar impairments in TAPSE and RV S’ among those with RV dysfunction (*P* < .001 for both).

**TABLE 2:** Imaging characteristics

	Overall (n = 101)	RV Dysfunction+ (n = 31)	RV Dysfunction− (n = 70)	*P*
Cardiac morphology/function/tissue properties				
CMR (Left ventricle)				
Ejection fraction (%)	42.6 ± 15.3	29.9 ± 9.2	48.3 ± 14.1	<.001
End‐diastolic volume (mL)	201.9 ± 63.6	233.9 ± 58.3	187.8 ± 60.9	.001
End‐systolic volume (mL)	122.0 ± 62.2	166.5 ± 53.0	102.3 ± 55.8	<.001
CMR (Right ventricle)				
Ejection fraction (%)	52.4 ± 11.6	38.5 ± 8.0	58.7 ± 6.4	<.001
End‐diastolic volume (mL)	151.2 ± 52.1	176.6 ± 57.2	139.8 ± 45.7	.001
End‐systolic volume (mL)	75.1 ± 41.5	110.9 ± 49.0	59.0 ± 24.5	<.001
Echo (Left ventricle)				
Ejection fraction (%)	41.8 ± 15.5	29.1 ± 9.0	47.5 ± 14.4	<.001
End‐diastolic diameter (cm)	5.9 ± 0.6	6.2 ± 0.5	5.8 ± 0.6	.001
Pulmonary arterial pressure (mm Hg)	38.5 ± 15.2	45.1 ± 16.0	35.3 ± 13.9	.006
Pulmonary hypertension^a^	42% (42)	73% (19)	43% (23)	.01
Manual and machine learning‐derived RV function				
Manual				
Lateral				
LTAD (cm)	3.2 ± 1.0	2.8 ± 0.8	3.4 ± 1.0	<.001
CTAD (cm)	8.5 ± 3.2	7.4 ± 3.3	9.0 ± 3.0	<.001
Septal				
LTAD (cm)	1.6 ± 0.6	1.3 ± 0.5	1.7 ± 0.6	<.001
CTAD (cm)	4.9 ± 2.1	4.2 ± 2.1	5.3 ± 2.1	<.001
Machine learning				
Lateral				
LTAD (cm)	3.1 ± 1.0	2.6 ± 0.7	3.3 ± 1.1	<.001
CTAD (cm)	8.5 ± 4.0	7.0 ± 2.9	9.1 ± 4.3	<.001
Septal				
LTAD (cm)	1.6 ± 0.9	1.2 ± 0.5	1.8 ± 1.0	<.001
CTAD (cm)	4.5 ± 2.7	3.4 ± 1.6	4.9 ± 3.0	<.001
Conventional				
TAPSE (cm)	1.8 ± 0.4	1.5 ± 0.3	1.9 ± 0.4	<.001
S′ (cm/s)	11.2 ± 2.8	9.5 ± 2.4	12.0 ± 2.6	<.001
FAC (%)	36.5 ± 10.4	29.3 ± 8.6	40.2 ± 9.4	<.001

Note

Data presented as mean ± SD (data in parentheses refer to range for each respective variable). RV dysfunction: RVEF < 50%.

^a^ Pulmonary hypertension defined as PASP > 35 mm Hg.

### 3.2 Comparison between manual and automated machine learning segmentation

**FIGURE 3:**
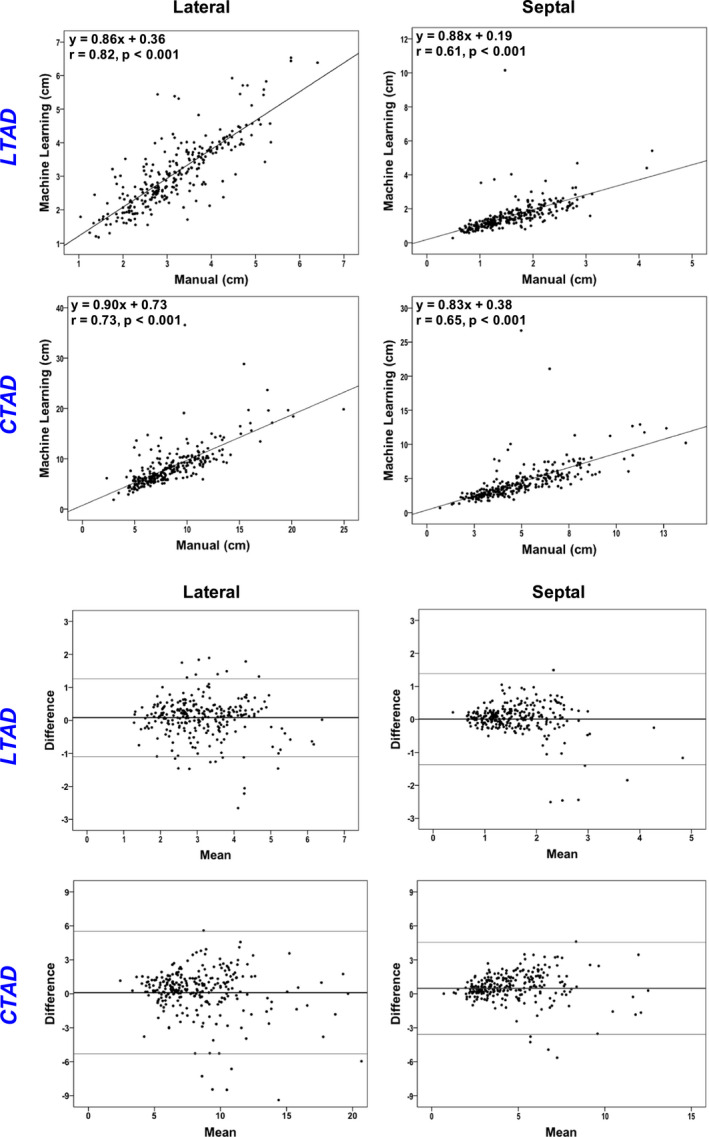
Logistic regression and Bland–Altman analyses comparing automated and manual annular segmentation. Note good correlations (*r *= .61–.82) and reasonable limit of agreements for both LTAD and CTAD (LOA‐1.09‐1.39 and −5.3‐5.5, respectively). CTAD = circumferential tricuspid annular displacement; LTAD = linear tricuspid annular displacement

When comparing manual and automated quantification of annular segmentation, all displacement correlations were good (*r* = .61–.82) with reasonable limit of agreement for both (−1.09 to 1.39 and −5.31 to 5.50, respectively). Scatter plot and Bland–Altman analyses using ML‐derived annular tracking in relation to manual quantification are shown in Figure 3.

To assess reproducibility, manual and automated lateral LTAD and CTAD quantification were performed in 22% of random subset of studies (Table 3). Reproducibility was, unsurprisingly, high for ML algorithm with zero inter‐ and intra‐observer variability and intra‐class correlation coefficient of 1.0. Inter‐ and intra‐observer reproducibility of manual segmentation was lower as compared to automated segmentation, but overall demonstrated high intra‐class correlation coefficient and small limits of agreement (0.87–0.91 and −4.46 to 3.46).

**TABLE 3:** Reproducibility analysis for lateral annular displacement

	Mean ± SD [cm]	(LOA) [cm]		ICC	*P*
Inter‐observer					
LTAD	0.30 ± 0.50	(−0.68, 1.29)		0.89 (0.73‐0.95)	<.001
CTAD	0.52 ± 1.59	(−2.41, 3.46)		0.91 (0.84‐0.95)	<.001
Intra‐observer					
LTAD	−0.32 ± 0.55		(−1.39, 0.75)	0.87 (0.69‐0.93)	<.001
CTAD	−1.10 ± 1.71		(−4.46, 2.26)	0.88 (0.69‐0.94)	<.001

### 3.3 Diagnostic performance for RV function

**FIGURE 4:**
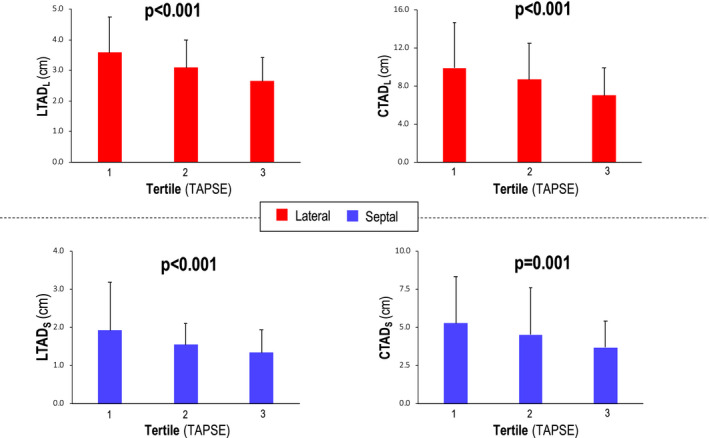
Tricuspid annular segmentation in relation to population‐based tertiles of TAPSE. As shown, both lateral and septal linear tricuspid annular displacement (left) as well as lateral and septal circumferential tricuspid annular displacement (right) decreased stepwise in relation to TAPSE strata. CTAD_L_ = lateral circumferential tricuspid annular displacement; CTAD_S_ = septal circumferential tricuspid annular displacement; LTAD_L_ = lateral linear tricuspid annular displacement; LTAD_S_ = septal linear tricuspid annular displacement

**FIGURE 5:**
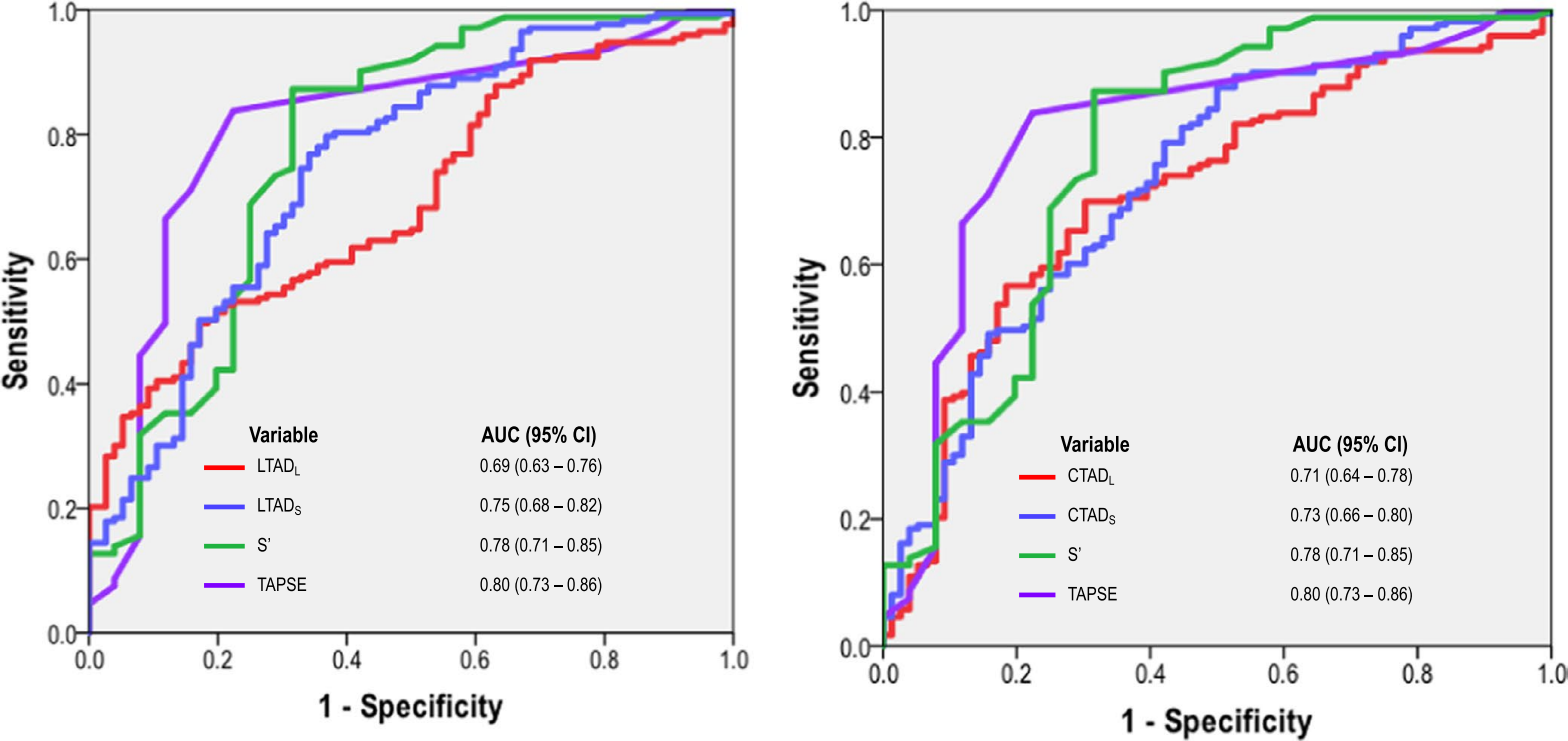
Receiver operating characteristic curve for automated and conventional RV quantification in relation to RVEF < 50% as established reference standard of CMR. Note automated and conventional indices yielded similar diagnostic performance assessed as area under the curve. CMR = cardiac magnetic resonance; RV = right ventricle; RVEF = right ventricle ejection fraction

As shown in Figure 4, both LTAD and CTAD decreased stepwise in relation to population‐based tertiles of TAPSE, with similar results when ML analyses were localized to the septal or lateral tricuspid annulus (all *P* ≤ .001). Figure 5 demonstrates RV annular segmentation techniques developed in this study to yield good diagnostic performance for discriminating RV dysfunction defined by CMR. Automated ML‐derived approach had good overall performance in relation to CMR defined RV dysfunction (AUC 0.69–0.73), which were overall slightly lower as compared to conventional RV annular indices quantified as TAPSE and *S*′ (0.78–0.80). Manual and ML‐derived RV annular shortening indices were tested with regard to diagnostic performance for RV dysfunction as defined by CMR. Applying ML‐derived annular cutoffs to maximize sensitivity (>80%), findings yielded comparable high negative predictive value (84%–87%) but lower positive predictive value (37%–40%) in relation to conventional RV indices, TAPSE and RV *S*′ (NPV 83%–88%, PPV 64%–66%; Table4). Applying the same optimized cutoffs, manual segmentation of RV annulus also yielded similar diagnostic performance (NPV 83%–87%, PPV 37%–39%).

**TABLE 4:** Test performance characteristics

	AUC (95% CI)	Cutoff (%)	Sensitivity (%)	Specificity (%)	Accuracy (%)	PPV (%)	NPV (%)
Machine Learning							
Lateral							
LTAD	0.69 (0.63‐0.76)	3.41	84	44	56	40	86
CTAD	0.71 (0.64‐0.78)	8.93	85	45	57	40	87
Septal							
LTAD	0.75 (0.68‐0.82)	1.75	83	39	52	37	84
CTAD	0.73 (0.66‐0.80)	4.81	83	43	55	39	85
Manual							
Lateral							
LTAD	0.68 (0.61‐0.75)	3.59	86	40	54	39	87
CTAD	0.70 (0.63‐0.78)	9.59	86	40	54	39	87
Septal							
LTAD	0.72 (0.65‐0.79)	1.78	80	42	54	38	83
CTAD	0.70 (0.63‐0.78)	5.61	81	40	52	37	83
Conventional							
TAPSE	0.80 (0.73‐0.86)	1.6	74	83	80	66	88
*S*′	0.78 (0.71‐0.85)	9.5	57	86	78	64	83

## 4 DISCUSSION

The primary aim of this study was to develop and validate a ML‐derived automated echo quantification methodology for RV functional assessment using 2D echocardiography. To the best of our knowledge, this is the first study to systematically test a deep learning–derived automated assessment of RV systolic function on 2D echo with CMR as the reference standard. Our ML‐derived segmentation algorithm successfully analyzed all cases with minimal processing time (<1 second) with no user editing required. Taken together, findings of this study demonstrate fully automated tricuspid annular displacement derived from a novel deep learning model to perform similarly to manual echo indices for the detection of CMR‐evidenced RV dysfunction, providing proof of concept regarding the feasibility of automated ML for 2D echo assessment of the RV.

Artificial intelligence, specifically ML, is currently being applied in medicine with the promise of providing physicians with a novel method of accurately and efficiently interpreting large amounts of quantitative information.^16^ ML applied to cardiac imaging can enable automated recognition and segmentation of cardiac structures and assist in the diagnosis of disease states. Knackstedt et al demonstrated that the left ventricular ejection fraction and longitudinal strain could be accurately and reproducibly computed from echo data using ML‐enabled software.^17^ Zhang et al trained convolutional neural networks to obtain measurements of the left ventricle and predict various disease states including hypertrophic cardiomyopathy, cardiac amyloidosis, and pulmonary arterial hypertension.^8^ While these studies demonstrate feasibility for utilization of ML approaches for LV functional assessment, there are limited data for its use for RV assessment, likely owing to the geometric complexity of RV structure.

More recently, ML‐based 3D echo algorithm to quantify RV volumes and RV ejection fraction was tested in a retrospective cohort.^9^ Although quantification was feasible in all fifty‐six patients, the automatic approach was only accurate in 32% of the study population. Endocardial contour editing was necessary in the remaining 68% of patients and resulted in a sevenfold increase in processing time. In addition, while 3D echo is an excellent RV quantification methodology, it should be noted that obtaining optimal 3D echo image acquisition can be challenging and time‐consuming. As such, 2D echo is the most widely used screening tool to assess RV structure and function. In this regard, our findings support that it is possible to successfully automate assessment of RV function on conventional 2D echo, creating a robust and readily available solution with its application particularly attractive for large‐scale population‐based studies.

Our findings should be noted in the context of the following limitations. The study population included 101 CAD patients from a single institution, and although automated measurements were reliable and comparable to manual measurements, they did not provide substantially higher diagnostic utility. It is important to note that while equivalent views for conventional and aML segmentation were utilized whenever available for measurement, it is possible that slight variations in transducer angulation and resultant views could have yielded differences in displacement values between conventional aML segmentation. It is also possible that visualization of cardiac structure and function itself could have led to reader bias when in assessing TAPSE and *S*′. Such bias could contribute to higher diagnostic performance of conventional versus automated measurements. In this context, it is also important to note that TAPSE itself is not without limitation, as suboptimal placement of the M‐mode cursor can result in angle‐dependent inaccuracy of RV function. Automated annular segmentation has the potential to overcome this limitation. These concepts need further testing within the framework of a larger population with wider range of RV function and further training, which itself has the potential to improve diagnostic performance. Future machine learning techniques could also include an ensemble of models evaluating several parameters (eg, annular displacement, strain, TAPSE, *S*′) as opposed to a single measurement, which has the potential to further improve its diagnostic performance.

In conclusion, fully automated tricuspid annular displacement from a novel deep learning model performs well in relation to manual echo indices for the detection of CMR‐evidenced RV dysfunction. This study adds to the growing literature that ML‐based algorithms can improve image interpretation efficiency and reliability and is the first of its kind to systematically test and validate ML‐derived 2D RV indices. Further research is warranted to test diagnostic and prognostic utility of ML‐derived tricuspid annular displacement in large population‐based cohorts.

## CONFLICTS OF INTERESTS

None.

## References

[1] van Wolferen SA , Marcus JT , Boonstra A , et al. Prognostic value of right ventricular mass, volume, and function in idiopathic pulmonary arterial hypertension. Eur Heart J. 2007;28:1250–1257. 17242010 10.1093/eurheartj/ehl477

[2] Davlouros PA , Niwa K , Webb G , et al. The right ventricle in congenital heart disease. Heart. 2006;92(Suppl 1):i27–i38. 16543599 10.1136/hrt.2005.077438 PMC1860730

[3] Pueschner A , Chattranukulchai P , Heitner JF , et al. The prevalence, correlates, and impact on cardiac mortality of right ventricular dysfunction in nonischemic cardiomyopathy. JACC Cardiovasc Imaging. 2017;10:1225–1236. 29025576 10.1016/j.jcmg.2017.06.013

[4] Ghio S , Recusani F , Klersy C , et al. Prognostic usefulness of the tricuspid annular plane systolic excursion in patients with congestive heart failure secondary to idiopathic or ischemic dilated cardiomyopathy. Am J Cardiol. 2000;85:837–842. 10758923 10.1016/s0002-9149(99)00877-2

[5] Kim J , Srinivasan A , Seoane T , et al. Echocardiographic linear dimensions for assessment of right ventricular chamber volume as demonstrated by cardiac magnetic resonance. J Am Soc Echocardiogr. 2016;29:861–870. 27297619 10.1016/j.echo.2016.05.002 PMC5057385

[6] Haddad F , Hunt SA , Rosenthal DN , Murphy DJ . Right ventricular function in cardiovascular disease, part I: anatomy, physiology, aging, and functional assessment of the right ventricle. Circulation. 2008;117:1436–1448. 18347220 10.1161/CIRCULATIONAHA.107.653576

[7] Sheehan F , Redington A . The right ventricle: anatomy, physiology and clinical imaging. Heart. 2008;94:1510–1515. 18931164 10.1136/hrt.2007.132779

[8] Zhang J , Gajjala S , Agrawal P , et al. Fully automated echocardiogram interpretation in clinical practice. Circulation. 2018;138:1623–1635. 30354459 10.1161/CIRCULATIONAHA.118.034338 PMC6200386

[9] Genovese D , Rashedi N , Weinert L , et al. Machine learning‐based three‐dimensional echocardiographic quantification of right ventricular size and function: validation against cardiac magnetic resonance. J Am Soc Echocardiogr. 2019;32:969–977. 31174940 10.1016/j.echo.2019.04.001

[10] Rudski LG , Lai WW , Afilalo J , et al. Guidelines for the echocardiographic assessment of the right heart in adults: a report from the American Society of Echocardiography endorsed by the European Association of Echocardiography, a registered branch of the European Society of Cardiology, and the Canadian Society of Echocardiography. J Am Soc Echocardiogr. 2010;23:685–713; quiz 786–8. 20620859 10.1016/j.echo.2010.05.010

[11] Han X . MR‐based synthetic CT generation using a deep convolutional neural network method. Med Phys. 2017;44:1408–1419. 28192624 10.1002/mp.12155

[12] Ronneberger O , Fischer P , Brox T . U‐Net: convolutional networks for biomedical image. Segmentation. 2015;234–241.

[13] He K , Zhang X , Ren S , Sun J "Deep Residual Learning for Image Recognition," 2016 IEEE Conference on Computer Vision and Pattern Recognition (CVPR) (pp. 770–778). Las Vegas, NV 2016.

[14] Bland J , Altman D . Statistical methods for assessing agreement between two methods of clinical measurement. Lancet. 1986;i:307– 310. *Web copy of Bland and Altman*. 2868172

[15] Jones E , Oliphant T , Peterson P . SciPy: Open source scientific tools. for Python; 2001.

[16] Johnson KW , Torres Soto J , Glicksberg BS , et al. Artificial Intelligence in Cardiology. J Am Coll Cardiol. 2018;71:2668–2679. 29880128 10.1016/j.jacc.2018.03.521

[17] Knackstedt C , Bekkers SCAM , Schummers G , et al. Fully automated versus standard tracking of left ventricular ejection fraction and longitudinal strain: the FAST‐EFs multicenter study. J Am Coll Cardiol. 2015;66:1456–1466. 26403342 10.1016/j.jacc.2015.07.052

